# Evaluating the Optimal Operating Table Height for ProSeal-LMA™ Insertion

**DOI:** 10.1155/2022/5118362

**Published:** 2022-11-17

**Authors:** Song Lin Low, Azlina Masdar, Nadia Md. Nor, Azrin Mohd Azidin, Hsueh Jing Low, Siti Nidzwani Mohamad Mahdi

**Affiliations:** ^1^Department of Anaesthesiology and Intensive Care, Universiti Kebangsaan Malaysia Medical Centre, Jalan Yaacob Latif, Bandar Tun Razak, Cheras 56000, Kuala Lumpur, Malaysia; ^2^Department of Anaesthesiology and Intensive Care, Hospital Tuanku Jaafar, Seremban 70300, Malaysia; ^3^Department of Anaesthesia and Intensive Care, Hospital Kuala Lumpur, Jalan Pahang 50586, Kuala Lumpur, Malaysia

## Abstract

**Introduction:**

ProSeal-Laryngeal Mask Airway™ (P-LMA™) is one of the commonly used laryngeal mask airways. Despite the proper insertion technique, suboptimal positioning and airway morbidity still occurs. This study explored the possibility of the operating table height position affecting successful P-LMA™ placement.

**Methods:**

A total of 138 patients aged between 18 and 65 years old with the American Society of Anesthesiologists (ASA) I or II status, who required general anaesthesia and had no contraindication towards the use of P-LMA™, were recruited. They were randomly positioned into three anatomical landmarks, which were umbilicus, lowest rib margin, and xiphoid. P-LMA™ was inserted following muscle paralysis, and the first successful placement was evaluated using positional and performance tests. Duration, ease of P-LMA™ insertion, and airway complications were compared.

**Results:**

Demographic and airway features were comparable among all groups. The P-LMA™ placement success rate improved when the table height was positioned at the lowest rib margin (*p*=0.002). All three positions were comparable in terms of duration, ease of insertion, and airway morbidities.

**Conclusion:**

The lowest rib margin anatomical landmark can be used as a guide in achieving the optimal operating table height for successful P-LMA™ placement.

## 1. Introduction

Airway management is a crucial period during the administration of general anaesthesia where the focus and stress level of the attending anaesthesiologists are at their highest [1]. In 1983, Dr. Archie Brain successfully developed the world's first laryngeal mask airway (LMA) [2]. ProSeal-Laryngeal Mask Airway™ (P-LMA™) is a second-generation LMA that is commonly used as it provides numerous advantages when compared to endotracheal intubation, including superior haemodynamic stability, less airway trauma, and superior patients' comfort [3–5]. The success rate of P-LMA™ insertion ranges from 77% to 90%, and suboptimal positioning occurs in 30–66% of cases [6–8]. Airway morbidity still occurs even with the apparent proper insertion of P-LMA™ [9–11]. Therefore, P-LMA™ placement quality may be affected by factors other than device failure, operator experience, and the operator's technique of insertion.

As such, the LMA should be inserted and placed in an optimal position to minimize complications and allow for maximal functionality [12]. Lee et al. stated that the table height at the xiphoid level provides an optimal laryngeal view during endotracheal intubation [13]. However, whether this factor has the same effect on LMA insertion is not yet known. To the best of our knowledge, no similar study was conducted on investigating the optimal table height for P-LMA™ placement. As the usage of LMA has gained much importance, especially as a rescue device for failed intubation [14], we studied the optimal operating table height which could allow for a successful insertion of P-LMA™ at the first attempt. Factors such as duration taken and ease of insertion, as well as the associated complications, were also evaluated.

## 2. Materials and Methods

This prospective, randomised clinical trial was conducted from August 2019 to July 2020 after obtaining institutional ethics committee approval (Research no: FF-2019-179; Clinicaltrials.gov identifier: NCT04338412) and patients' written informed consent. Patients recruited were between 18 and 65 years old with the American Society of Anesthesiologists (ASA) I or II status and required elective or emergency surgeries under general anaesthesia, where usage of P-LMA™ was not contraindicated. Patients who had clinical features of the difficult airway (Mallampati III or IV, mouth opening less than three finger breadths, or thyromental distance (TMD) less than three finger breadths), congenital or acquired airway abnormality, cervical spine pathology, edentulous or loose tooth, and body mass index (BMI) ≥ 35 kg/m^2^, as well as intra-abdominal and laparoscopic surgeries were excluded from the study. The dropout criteria were as follows: surgical case cancellation, inability to insert P-LMA™, and unexpected difficult bag-mask ventilation. P-LMA™ insertion was conducted by multiple anaesthesiology trainees, all with more than three years of anaesthetic experience.

**Figure 1:**
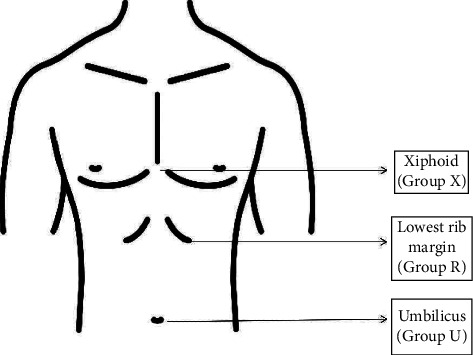
Height of the operating table corresponding to the operator's umbilicus (Group U), lowest rib margin (Group R), or xiphoid process (Group X).

**Figure 2:**
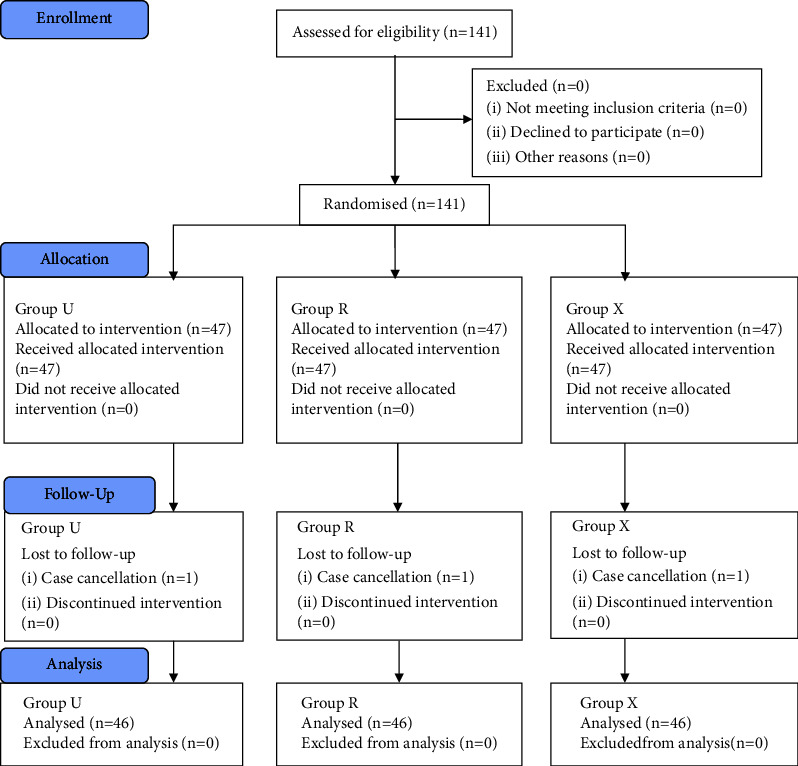
CONSORT flowchart.

All patients were recruited by the primary investigator during the preoperative assessment. Patients' demographic characteristics, as well as airway assessment consisting of the Mallampati score, mouth opening, and TMD, were recorded preoperatively. Patients were required to fast for at least six hours before the scheduled operation. They were randomised using a computer-generated randomisation method into three groups based on the height of the operating table, for which these levels correspond to the anaesthesiology trainee's umbilicus (Group U), lowest rib margin (Group R), or xiphoid process (Group X), as shown in Figure 1 and the CONSORT flowchart in Figure 2.

In the operating theatre, standard intraoperative monitoring such as electrocardiogram, pulse oximetry, noninvasive blood pressure measurement, and capnography were applied to all subjects. Patients were preoxygenated with 100% oxygen until end-tidal oxygen of 85% was achieved. Intravenous (IV) anaesthetic-inducing drugs were then administered: fentanyl 1.5 mcg/kg and propofol 2.0 mg/kg. After loss of consciousness, the attending anaesthesiology trainee proceeded with mask ventilation with a mixture of oxygen and sevoflurane to achieve a minimum alveolar concentration (MAC) of 1.0–1.2. If there was no difficulty in bag-mask ventilation, IV rocuronium 0.6 mg/kg was administered, and the patient was subsequently ventilated for another three minutes. If unexpected difficult bag-mask ventilation occurred, the patient was excluded from the study, and subsequent airway management was at the discretion of the attending anaesthesiologist, guided by the Difficult Airway Society guidelines.

After the patient was adequately paralysed, the operating table height was adjusted based on the assigned intervention, with the patients' forehead used as a point of reference to correlate with the anaesthesiology trainee's anatomical landmark. The P-LMA™ was then inserted by the attending anaesthesiology trainee. The choice of P-LMA™ size was based on the manufacturer's recommendation. All P-LMA™ were manufactured by The Laryngeal Mask Airway Co., Ltd., Mahe, Seychelles, with compliance to its recommended usage of up to 40 times and sterilised between uses by autoclaving [15].

All P-LMA™ were inserted with cuff fully deflated using the introducer technique with patients' head in “sniffing the morning air” position. Subsequently, P-LMA™ cuffs were inflated with a volume of air recommended by the manufacturer. A cuff pressure manometer was used to ensure that an intracuff pressure of 60 cm H_2_O was achieved.

The duration taken for P-LMA™ insertion was timed by using a standard stopwatch, beginning from the time when the anaesthesiology trainee placed the P-LMA^TM^ at the aperture of the oral orifice to the appearance of the first end-tidal CO_2_ waveform. Time measurement was only performed during the first attempt at P-LMA™ insertion.

The placement of P-LMA™ was considered successful when it fulfilled both positional and performance tests [3, 4, 9]. Positional tests included the gastric tube “bubble” test, suprasternal notch tap test, and insertion of a gastric tube through its drainage tube, which verified the ideal position of P-LMA™ placement [3, 4, 9]. The performance test used was the oropharyngeal leak pressure test, which determined the functionality of P-LMA™ after insertion [3, 4, 15, 16]. The outcomes of these tests were recorded by a second anaesthesiology trainee who was not involved in the insertion process. Subsequent intraoperative general anaesthesia management was carried out by the attending anaesthesiologist.

A gastric tube “bubble” test was performed by sealing the gastric drainage tube with a drop of gel 2-3 mm height followed by intermittent positive pressure ventilation with a set tidal volume of 8 ml/kg. If no air leaks out and gel remains in the drainage tube, it suggests good P-LMA™ placement. Consequently, with the gel remaining intact, a suprasternal notch test was performed by placing firm pressure on the suprasternal notch with a finger. P-LMA™ placement is considered good if the gel column moves synchronously with the applied pressure. After that, a 14-Fr or 16-Fr gastric tube was inserted via the gastric drainage tube. Successful smooth gastric tube insertion was verified by the presence of gastric fluid aspiration and audible gastric insufflation.

Subsequently, to test for an oropharyngeal leak, airway seal pressure was determined by setting the adjustable pressure limiting (APL) valve to 30 cm H_2_O and a fixed fresh gas flow rate of 3 L/min. A good oropharyngeal leak pressure was determined by its capability to record a stable airway pressure of ≥25 cm H_2_O in the supine position. If all tests were fulfilled, it was indicative of successful P-LMA™ placement.

Failure of P-LMA™ placement during the first attempt, defined as any failure of individual positional or performance tests, was considered failed P-LMA™ attempt, and subsequent airway management was at the discretion of the attending anaesthesiologist.

Ease of insertion was evaluated by the anaesthesiology trainee via subjective ease of the insertion score: 1 = no resistance, 2 = minimal resistance, 3 = moderate resistance, and 4 = unable to place the device [17]. The presence of airway morbidity and complications including oxygen desaturation of <90%, airway obstruction, laryngospasm, bronchospasm, and oropharyngeal trauma (defined as the presence of blood upon removal of P-LMA™) were recorded. Upon completion of the procedure, the operating table height was adjusted according to the surgical operation requirement.

### 2.1. Statistical Analysis

The required sample size was calculated using the G^*∗*^Power software (version 3.1.9.2) with an ANOVA input. The alpha value was set at 0.05, and the sample size was calculated based on the result of a pilot study. The largest sample size computed among all our objectives was based on the duration taken for P-LMA™ insertion, with an effect size from the mean, *f* was 0.35. A total of 150 subjects were required for this study to achieve 80% power of the study, including a 10 percent dropout rate.

The data were analysed using SPSS (Statistical Package for the Social Sciences) for Windows version 25.0 software (IBM Corp, Armonk, NY, USA). The results were presented as a mean ± standard deviation, median (interquartile range), or frequency (percentages), where applicable. For intergroup analysis, the analysis of variance (ANOVA) test and the Kruskal–Wallis test were used for normally distributed continuous data and nonnormally distributed data, respectively. Post hoc analyses were conducted by using Bonferroni correction for multiple hypotheses testing in intergroup analysis. Qualitative data analysis was conducted using the chi-square or Fisher exact test if the assumption was not met. A *p* value < 0.05 was considered statistically significant.

## 3. Results

One hundred and forty-one patients were recruited, with three patients dropped out from the study due to surgical cancellation, making the total number of subjects 138. Demographic data were comparable, as shown in Table 1. The patients' preanaesthetic airway assessment and anaesthesiology trainees' height were also similar between the three groups (Table 2).

The overall success rate of all three groups' first attempt P-LMA™ insertion was 60% (Table 3). We found that Group R had a greater statistically significant success rate when compared to both Group U and Group X (*p*=0.002, Table 3), which was confirmed with further post hoc analysis. Individual analysis of positional and performance tests also showed a statistical difference for Group R vs. Group X, with further validation by post hoc analysis for the suprasternal notch test (*p*=0.007), gastric tube insertion test (*p*=0.009), and peak airway pressure test (*p*=0.008).

There was no significant difference in the median duration of P-LMA™ placement (*p*=0.236) and ease of insertion (*p*=0.105) (Table 4). Occurrences of traumatic P-LMA™ insertion were comparable between the three groups. No other airway morbidities, such as desaturation, airway obstruction, laryngospasm, and bronchospasm, were documented throughout this study.

## 4. Discussion

The first attempt of P-LMA™ insertion of all three positions in the present study yielded a 60% success rate. This was in contrast to previous studies, which were successful in 77–90% of the attempts [6–8]. The disparities in these findings may have been related to the varying fulfilment/success criteria that had to be achieved before considering P-LMA^TM^ insertion to be successful. The present study required all four clinical tests to be successful, which was higher than in past studies.

Further evaluation of the effect of the table height on the insertion success rate revealed a significant improvement when P-LMA™ insertion was performed at the level of the lowest rib margin when compared to the xiphoid and umbilicus positions. This was in contrast to those reported by Lee et al., who showed xiphoid as the optimal operating table height for tracheal intubation [13]. Based on the ergonomic design for a standing workplace, an ideal table height is within the range of 10 cm above the elbow level to 15 cm below it, which falls within the range of all three positions tested in this study [18]. However, the xiphoid and umbilicus positions are at the extreme end of the ergonomic working range. At the xiphoid position, anaesthesiology trainees were required to work beyond these ergonomic levels, possibly accounting for a higher failure rate of P-LMA™ placement.

Suboptimal head positioning could lead to improper oropharyngeal angulation, leading to difficult or failed P-LMA™ insertion [19]. As the P-LMA™ insertion success rate at xiphoid and umbilical positions was lower, suboptimal head positioning could have occurred, leading to a failure of proper placement. This may be confounded by the inflexible angle of insertion due to the usage of the rigid P-LMA™ introducer [20].

Positional and performance tests play an essential role in the confirmation of P-LMA™ placement [3, 4, 9]. In the post hoc analysis, comparing the lowest rib margin and the xiphoid position, we found statistically significant variations among findings in the suprasternal notch, gastric tube insertion, and peak airway pressure tests. The primary objective of the suprasternal notch test is to detect overfolding of the P-LMA™ tip, which is indicated by the absence of synchronous movement of the gel column in the gastric drainage tube with transmitted pressure. However, the suprasternal notch test result can also be affected by diminished pressure transmission as a result of the open oesophagus, folded tip of P-LMA™ with incomplete obstruction of the gastric drainage tube, tapping over the anterolateral neck or pilot balloon and positive pressure ventilation [21]. This further complicates the role of the suprasternal notch test in confirming the optimal placement of P-LMA™. As such, numerous studies advocated reinforcing positional tests with the gastric tube insertion test [9, 21]. An easy insertion of a gastric tube through P-LMA™'s gastric drainage tube excludes the possibility of a twisted or folded tip [9, 21]. The present study showed a significantly higher success rate for both the suprasternal notch and gastric tube insertion test at the lowest rib margin position. We postulated that umbilical and xiphoid positions contributed to the suboptimal angle of P-LMA™ insertion, resulting in a higher incidence of folding of the P-LMA™ tip.

In the case of the performance test, the oropharyngeal leak pressure test was performed to determine the maximum achievable airway pressure before air leaks, reflecting P-LMA™ ability for positive pressure ventilation and prevention of gastric insufflation [3, 4]. A standard intracuff pressure of 60 cm H_2_O was used throughout the present study. However, failed peak airway pressure tests do not always accompany audible leaks. This was highlighted by the lower presence of audible leaks than the number of those who failed the peak airway pressure test in the present study. We hypothesise that differences in the patients' upper airway soft tissue structure may be the underlying cause for this discrepancy. It is also plausible that the lowest rib margin position resulted in a better P-LMA™ placement quality, leading to a lower number of audible leaks. In this study, the maximum minute ventilation test was not included as part of the performance test as intra-abdominal and laparoscopic surgeries were excluded.

Duration of insertion and occurrence of traumatic LMA insertion were comparable in all three groups, probably due to equivocal proficiency among anaesthesia trainees. The unique design of P-LMA™ makes it easy to use, even in a challenging situation such as the prone position [22, 23]. This is proven by our comparable ease of insertion results among all three groups in the present study.

Several limitations could have potentially affected the result of this study. The umbilicus and lowest rib margins have an uncertain relationship with one another in obese operators. Second, ease of insertion scoring was a subjective parameter. Despite the ease of an insertion score of one or two, P-LMA™ placement might not have been successful. We did not include fibreoptic evaluations for failed P-LMA™ placement as this study did not investigate the cause of failed P-LMA™ placement. Moreover, our focus was to determine P-LMA™'s clinical performance and functionality. Nevertheless, adding video laryngoscopy to confirm P-LMA™ placement may be useful in future studies.

## 5. Conclusion

We conclude that the lowest rib margin provides an optimal operating table height for successful P-LMA™ placement, in terms of position and performance. It can be used as a guide in achieving the optimal operating table height for successful P-LMA™ placement for novices, as well as during difficult airway scenarios.

## Figures and Tables

**Table 1:** Demographic data.

Group	U (*n* = 46)	R (*n* = 46)	X (*n* = 46)	*P* value
Age (years)	42.4 (14.3), 95% CI [38.3, 46.5]	37.2 (11.7), 95% CI [33.8, 40.6]	42.1 (15.1), 95% CI [37.7, 46.5]	0.155
Weight (kg)	64.5 (10.5), 95% CI [61.5, 67.5]	66.9 (13.1), 95% CI [63.1, 70.7]	64.7 (12.8), 95% CI [61.0, 68.4]	0.582
BMI (kg m^−2^)	25.0 (3.5), 95% CI [24.0, 26.0]	25.0 (4.0), 95% CI [23.8, 26.2]	24.2 (4.2), 95% CI [23.0, 25.4]	0.529
Gender				
Male	10 (21.7)	17 (37.0)	21 (45.7)	0.051
Female	36 (78.3)	29 (63.0)	25 (54.3)	
ASA				
ASA I	25 (54.3)	33 (71.7)	30 (65.2)	0.215
ASA II	21 (45.7)	13 (28.3)	16 (34.8)	

Data were presented as mean (standard deviation) or the number of patients (percentage). CI, confidence interval; BMI, body mass index in kgm^−2^; ASA, American Society of Anesthesiologists classification.

**Table 2:** Preanaesthetic airway evaluation and anaesthesia trainees' height.

Group	U (*n* = 46)	R (*n* = 46)	X (*n* = 46)	*P* value
Mallampati				0.265
I	13 (28.3)	19 (41.3)	20 (43.5)	
II	33 (71.7)	27 (58.7)	26 (56.5)	
Height (m)	1.70 (1.65–1.78)	1.74 (1.64–1.78)	1.74 (1.65–1.78)	0.488

Data were presented as the number of subjects (percentage) or median (25^th^ percentile–75^th^ percentile).

**Table 3:** Comparing different operating table height positions in determining successful first attempt P-LMA^TM^ insertion and its related individual positional and performance test.

	U (*n* = 46)	R (*n* = 46)	X (*n* = 46)	*P* value	*P* value^#^
First attempt P-LMA™ insertion	24 (52.2)	37 (80.4)	22 (47.8)	0.002^*∗*^	U vs. R: 0.004
					U vs. X: 0.677
					R vs. X: 0.001
Gastric tube “bubble” test	44 (95.7)	43 (93.5)	39 (84.8)	0.236	U vs. R: 1.000
					U vs. X: 0.158
					R vs. X: 0.180
Suprasternal notch test	31 (67.4)	38 (82.6)	26 (56.5)	0.025^*∗*^	U vs. R: 0.092
					U vs. X: 0.283
					R vs. X: 0.007
Gastric tube insertion test	39 (84.8)	42 (91.3)	32 (69.6)	0.021^*∗*^	U vs. R: 0.335
					U vs. X: 0.082
					R vs. X: 0.009
Peak airway pressure test (≥25 cm H_2_O)	27 (58.7)	37 (80.4)	25 (54.3)	0.020^*∗*^	U vs. R: 0.023
					U vs. X: 0.674
					R vs. X: 0.008
No audible leak	40 (87.0)	43 (93.5)	36 (78.3)	0.105	U vs. R: 0.485
					U vs. X: 0.271
					R vs. X: 0.036

Data were presented as number of patients (percentage). ^*∗*^*P* value < 0.05 denotes statistical significance. ^#^*P* value < 0.0167 denotes statistical significance with Bonferroni correction.

**Table 4:** P-LMA™ insertion duration, ease of the insertion score, and traumatic insertion.

Group	U (*n* = 46)	R (*n* = 46)	X (*n* = 46)	*P* value
P-LMA™ insertion duration (seconds)	25.9 (23.1–32.6)	26.3 (21.5–31.3)	28.5 (22.4–35.6)	0.402

Ease of the insertion score				
1	20 (43.5)	23 (50.0)	17 (37.0)	0.259
2	20 (43.5)	21 (45.7)	20 (43.5)	
3	6 (13.0)	2 (4.3)	9 (19.6)	
4	0 (0)	0 (0)	0 (0)	

Traumatic LMA insertion	11 (23.9)	9 (19.6)	13 (28.3)	0.620

Data were presented as the number of subjects (percentage) or median (25^th^ percentile–75^th^ percentile).

## Data Availability

The data that support the findings of this study are available from the corresponding author upon request.
